# Use of estetrol with other steroids for attenuation of neonatal hypoxic-Ischemic brain injury: to combine or not to combine?

**DOI:** 10.18632/oncotarget.9591

**Published:** 2016-05-26

**Authors:** Ekaterine Tskitishvili, Christel Pequeux, Carine Munaut, Renaud Viellevoye, Michelle Nisolle, Agnes Noël, Jean-Michel Foidart

**Affiliations:** ^1^ Laboratory of Development Biology and Tumor, GIGA-Cancer, Department of Obstetrics and Gynecology/Department of Clinical Sciences, University of Liege, Liege 1, Belgium; ^2^ Neonatal Intensive Care Unit, Department of Pediatrics, University of Liege, Liege 1, Belgium; ^3^ Department of Obstetrics and Gynecology, University of Liege, Liege1, Belgium

**Keywords:** neonatal hypoxic-ischemic encephalopathy, hippocampus, cortex, estetrol, estradiol, Pathology Section

## Abstract

Estetrol (E4), estradiol (E2) and progesterone (P4) have important antioxidative and neuroprotective effects in neuronal system. We aimed to study the consequence of combined steroid therapy in neonatal hypoxic-ischemic encephalopathy (HIE). *In vitro* the effect of E4 combined with other steroids on oxidative stress and the cell viability in primary hippocampal cultures was evaluated by lactate dehydrogenase and cell survival assays. *In vivo* neuroprotective and therapeutic efficacy of E4 combined with other steroids was studied in HIE model of immature rats. The rat pups rectal temperature, body and brain weights were evaluated. The hippocampus and the cortex were investigated by histo/immunohistochemistry: intact cell number counting, expressions of markers for early gray matter lose, neuro- and angiogenesis were studied. Glial fibrillary acidic protein was evaluated by ELISA in blood samples. *In vitro* E4 and combinations of high doses of E4 with P4 and/or E2 significantly diminished the LDH activity and upregulated the cell survival.*In vivo*pretreatment or treatment by different combinations of E4 with other steroids had unalike effects on body and brain weight, neuro- and angiogenesis, and GFAP expression in blood. The combined use of E4 with other steroids has no benefit over the single use of E4.

## INTRODUCTION

Neonatal Hypoxic-ischemic Encephalopathy (HIE) is one of the primary reasons of severe impairment or death among preterm, near-term and term neonates. The most recent investigation is posing the speculation that neonatal HIE might start antenatally, implying importance of different factors (i.e. genetic and/or infectious, and placental factors), but parturition might have importance for the final development of HIE [1]. As a consequence, brain hypoxia along with ischemia and the reduced cerebral blood flow (CBF) might lead to perinatal HIE [2, 3]. The most vulnerable areas of the immature brain are: the cornu ammonis (CA1, CA3 and CA4) regions of the hippocampus, the cerebellum, layers III, V and VI of the neocortex and the neostriatum [4]. At cellular level different pathological cascades can directly contribute to long-term apoptosis in distal neuronal structures: oxidative stress, peroxynitrite-induced neurotoxicity, lipid peroxidation, mitochondrial and DNA damage [5-7]. Inflammatory mediators also have importance in the pathogenesis of HIE: NF-kB activation in neurons could provide survival, whereas activation in glial cells enhances neuronal cell death [8]. Inflammation may modulate vulnerability to brain damage and development of this pathological condition by representing a possible final common pathway of brain injury [9].

According to recent investigations, mortality and the neurodevelopmental outcomes in infants with moderate and severe HIE are tremendous: from 23 to 27% of infants die prior to discharge from the Neonatal Intensive Care unit (NICU), whereas from 37 to 38% die at follow up 18-22 months later [10, 11]. Among survivors, the neurodevelopmental outcome at 18 months might include: mental and psychomotor development retardation, cerebral palsy, epilepsy, blindness and hearing impairment [10, 11] though mild encephalopathy might result in understated changes in neurodevelopment later in life [12]. Different preclinical and clinical studies proposed several treatment possibilities for attenuation of HIE. Nowadays therapeutic hypothermia became the only strategy for treatment of HIE at term, and the rate of death or severe disability in infants with HIE is decreased from 60% to 46% after cooling [13].

Recent investigations already proved the neuroprotective efficacy of Estradiol (E2) and Progesterone (pregn-4-ene-3,20-dione) (P4) in different experimental models and clinical studies of neurological diseases: Parkinson's and Alzheimer's diseases, ischemic stroke, spinal cord injury, traumatic brain injury (TBI) and multiple sclerosis [14-25]. It was demonstrated that tremendous effect of P4 depends on attenuation of oxidative injury resulting from glutamate and glucose deprivation-induced toxicity [26-29]. It also protects against FeSO4 and amyloid β-peptide-induced toxicity *in vitro* in primary hippocampal cultures [30, 31].

E4 is a major E2 metabolite detected in maternal urine about 9 weeks of gestation, substantially increasing during pregnancy [32]. Our recent studies showed that E4 has very good antioxidant, neuroprotective, neuro- and angiogenic properties [33]. It was also shown that this steroid modulates the production of allopregnanolone in various brain regions, and upregulates expression of β-endorphin in the nervous system [34, 35]. E4 acts as selective estrogen receptor modulator (SERM) that activates the nuclear estrogen receptor α (ERα), but inhibits its membrane form. As a consequence, E4 has biological activities distinct from E2, depending on the tissues and cells, and the selective binding to the nuclear/membrane form of ERα [36]. Only limited data show the significance of separately used estrogens and progesterone in attenuation of neonatal hypoxic-ischemic encephalopathy [33, 37- 41] based on a model of HIE in a 7-day-old immature rats [42].

Postnatal E2 and P4 combined replacement in extremely preterm infants demonstrated reduction of the risk for cerebral palsy, spasticity, and ametropia at 5 years neurodevelopmental follow-up [43]. Several studies have suggested that P4 does not affect the positive effects of E2 [28, 44, 45], whereas others proposed that P4 might antagonize the positive effects of E2 [46-51].

Studies on neuroprotective properties of combined use of E4 with other steroids have not been performed so far. We aimed to study the possible effect of E4 with P4 and/or E2 for attenuation of neonatal HIE and define whether the combined use of E4 with other steroids might have any advantage over the single use of E4.

## RESULTS

### Effect of combined treatment of E4 alone or with E2 and/or P4 on H202-induced LDH activity and cell survival

**Figure 1:**
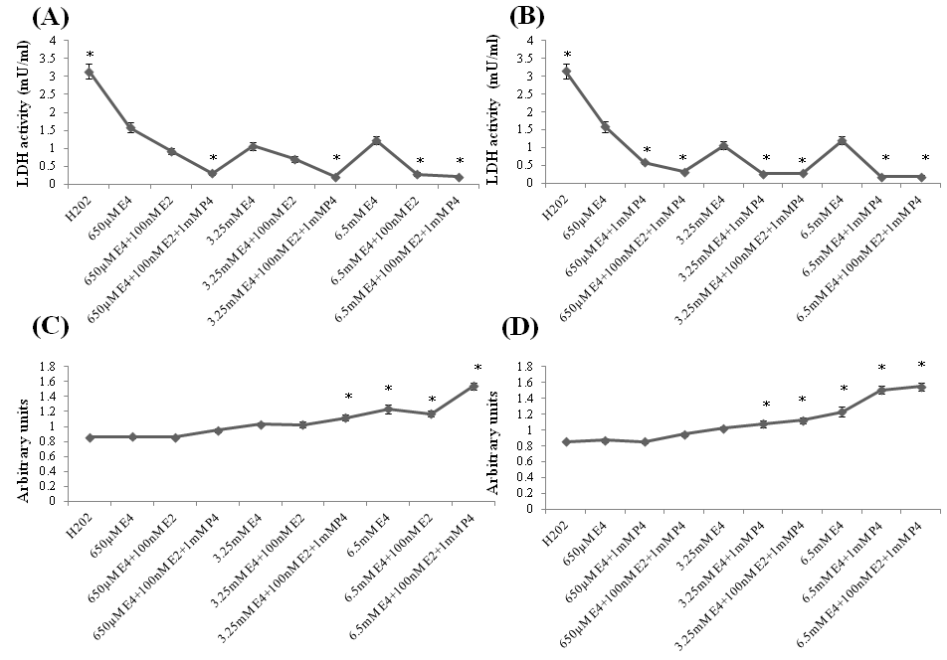
Effect of E4 alone or in combination with P4 and/or E2 on LDH activity and cell viability in primary hippocampal cell cultures subjected to the H202-induced oxidative stress **A.**-**D.** Primary hippocampal neuronal cells were treated with 650μM, 3.25mM and 6.5mM of estetrol alone or in combination with 100nM E2 and/or 1mM P4 for 1h after induction of oxidative stress by 100μM of H202 for 30min. **A.**-**B.** LDH activity was significantly downregulated in all the study groups compared to the H202-treated group. The LDH activity level was significantly lower in cultures treated either with 6.5mM E4 and 100nM E2 (A) or in cultures treated by any dose of E4 along with 1mM P4 than in cultures treated by E4 alone (B) as well as in cultures combinedly treated by any dose of E4 along with 1mM P4 and 100nM E2 than in cultures treated by E4 alone (A-B). **C.** Cell survival was significantly upregulated in cell cultures treated either by 6.5mM E4 with/without 100nM E2 or by 3.25mM, 6.5mM E4 with 100nM E2 and 1mM P4 than in H202-treated cultures. Cells exposed to 6.5mM E4 with/without 100nM E2 had significantly higher cell survival rate than the cultures treated by 650μM E4 with/without 100nM E2. Cells combinedly treated by 6.5mM E4 with 100nM E2 and 1mM P4 had significantly higher survival level than the cells treated by 6.5mM E4 with/without 100nM E2. **D.** Cell survival was significantly upregulated in cultures treated by 6.5mM E4 with/without 1mM P4 or by 3.25mM, 6.5mM E4 with 100nM E2 and 1mM P4 than in H202-treated cultures. The dose-dependent pattern was observed when 650μM, 3.25mM and 6.5mM E4 were used with/without 1mM P4. (C-D) Cell cultures combinedly treated by 6.5mM E4 with 100nM E2 and 1mM P4 had significantly higher survival level than the cells treated by 650μM, 3.25mM E4 in combination with 100nM E2 and 1mM P4. All measurements are expressed as mean±SEM.*p ≤ 0.05.

The treatment effect of E4 alone or combined with E2 and/or P4 on oxidative stress was studied in primary hippocampal cell cultures at 7 day *in vitro* (DIV). Cultures were exposed either to 650μM, 3.25mM and 6.5mM E4 alone or to combination of E4 with 100nM E2 and/or 1mM P4 by 1h after stimulation with 100μM of H202. As shown in (Figure 1A, 1B), LDH activity was significantly downregulated in all the study groups compared to the H202-treated group. The LDH activity level was significantly lower in cultures combinedly treated by different concentrations of E4 with E2 and P4 than in cultures treated by E4 alone (Figure 1A, 1B). Similar pattern of LDH activity was observed in cultures treated either by 6.5mM E4 with E2 (Figure 1A) or by different concentrations of E4 with P4 (Figure 1B) compared to cultures treated by E4 alone.

The cell survival rate was significantly increased in cultures treated either by 6.5mM E4 with/without E2 (Figure 1C) or by 6.5mM E4 with/without P4 (Figure 1D) or by high doses of E4 with E2 and P4 (Figure 1C, 1D) than in H202-treated cultures. Furthermore, cells exposed to 6.5mM E4 with/without E2 had significantly higher cell survival rate than the cultures treated by 650μM E4 with/without E2 (Figure 1C), though the dose-dependent pattern was more prominent when different concentrations of E4 were used with/without P4 (Figure 1D). Cells treated by 6.5mM E4 and P4 had significantly higher survival rate than the cells treated by E4 alone (Figure 1D). Cell cultures combinedly treated by 6.5mM E4 with E2 and P4 had significantly higher survival level than the cells treated either by 6.5mM E4 with/without E2 (Figure 1C) or by the lower doses of E4 combined with E2 and P4 (Figure 1C, 1D).

### Rat pups rectal temperature

**Figure 2:**
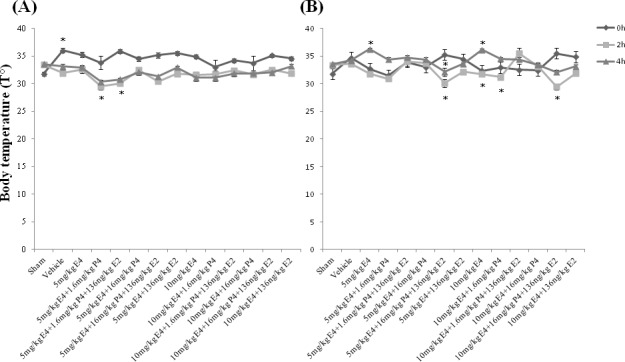
Post-operative rectal temperature and body weight of rat pups **A.** In neuroprotective model, immediately after hypoxic-ischemic (HI) insult (at 0 h), the rectal temperature was significantly increased only in the vehicle group than in the sham group, whereas 2h later the rectal temperature was significantly decreased in pretreated by 5mg/kg/day E4 and 1.6mg/kg/day P4 with/without 136ng/kg/day E2 groups compared to the sham group. 4h later no significant difference was observed among the study groups. **B.** In therapeutic model, between the study groups immediately after HI insult no significant differences were detected, whereas 2h later groups treated by combination of 5mg/kg/day or 10mg/kg/day E4 with 16mg/kg/day P4 plus 136ng/kg/day E2 had significantly decreased rectal temperature than the vehicle group or the groups treated by the same doses of E4 with 1.6mg/kg/day P4 and E2. Moreover, combination of 10mg/kg/day E4 with 16mg/kg/day P4 and 136ng/kg/day E2 significantly downregulated the rectal temperature compared to the sham group, or the group treated by 10mg/kg/day E4 and 16mg/kg/day P4 (Figure 2B). Also, the groups treated by 10mg/kg/day E4 alone or combined with 1.6mg/kg/day P4 had significantly decreased rectal temperature compared to the group treated by 10mg/kg/day E4 with 1.6mg/kg/day P4 and 136ng/kg/day E2. At 4h after HI event, animals treated by 5mg/kg/day or 10mg/kg/day E4 and 136ng/kg/day E2 with/without 16mg/kg/day P4 had significantly decreased rectal temperature along with the sham group compared to animals treated by single doses of E4 (Figure 2B). The same pattern was observed between groups treated by 10mg/kg/day E4 with 1.6mg/kg/day P4 and 136ng/kg/day E2, and the group treated by E4 alone. Treatment by 5mg/kg/day E4 with 16mg/kg/day P4 and 136ng/kg/day E2 significantly decreased the rectal temperature than the treatment by the same combination of compounds with 1.6mg/kg/day P4 (Figure 2B). All measurements are expressed as mean±SEM.*p ≤ 0.05.

Brain injury raises body temperature and the raised temperature is a marker for an underlying process with encephalopathy [52]. In neuroprotective model, immediately after hypoxic-ischemic (HI) insult (at 0h time point), the rectal temperature was significantly increased only in animals from the vehicle group compared to the sham group, whereas 2h later the rectal temperature was significantly decreased in groups pretreated by combination of 5mg/kg/d E4 and 1.6mg/kg/d P4 with/without 136ng/kg/d E2 compared to the sham group, though 4h later no significant differences were observed among the study groups (Figure 2A).

In therapeutic model, between the study groups immediately after HI insult no significant differences were detected, whereas 2h later groups treated by combination of any dose of E4 with 16mg/kg/day P4 plus 136ng/kg/d E2 had significantly decreased rectal temperature than the vehicle group or the groups treated by the same doses of E4 with 1.6mg/kg/d P4 and E2. Moreover, combination of 10mg/kg/d E4 with 16mg/kg/d P4 and 136ng/kg/d E2 significantly downregulated the rectal temperature compared to the sham group, or the group treated by the same doses of E4 and P4 (Figure 2B). Also, the groups treated by 10mg/kg/d E4 alone or combined with 1.6mg/kg/d P4 had significantly decreased rectal temperature compared to the group treated by the same doses of E4 and P4 along with E2. At 4h after HI event, animals treated by E4 and E2 with/without 16mg/kg/d P4 had significantly decreased rectal temperature along with the sham group compared to animals treated by single doses of E4 (Figure 2B). The same pattern was observed between groups treated by 10mg/kg/d E4 with 1.6mg/kg/d P4 and E2, and the group treated by E4 alone. Treatment by 5mg/kg/d E4 with 16mg/kg/d P4 and E2 significantly decreased the rectal temperature than the treatment by the same combination of compounds with 1.6mg/kg/d P4 (Figure 2B).

### Body and brain weight evaluation

Table 1 demonstrates that animals pretreated for 4 consecutive days by combination of 5mg/kg/d E4 and 16mg/kg/d P4 at postnatal day 7 (P7) had significantly higher weight than animals from the vehicle, sham and combinedly pretreated by 5mg/kg/d E4 and E2 groups. Brain-body weight ratio was significantly higher in groups pretreated by 5mg/kg/d E4 plus 136ng/kg/d E2 in combination either with 1.6mg/kg/d (0,054±0.001) or 16mg/kg/d P4 (0,054±0.001) than in the vehicles (0.042 ± 0.001). In treated groups only animals from 10mg/kg/d E4 group had significantly higher brain weight compared to the vehicles (Table 1) though the brain-body weight ratio was not significantly different between the study groups (data not shown).

**Table 1:** Body and Brain weights of rat pups from study groups

Groups		Body	Weight (g)	Brain	*P*
		P7	P14	weight (g)	
**Pretreatment**					
***Sham***		12.04±0.52	26.96±0.73	1.20±0.01	
***Vehicle***		12.52±0.41	27.86±0.65	1.15±0.01	
***5mg/kg E4***		15.03±0.68	24.61±1.19	1.18±0.03	
***5mg/kg E4+1.6mg/kg P4***		12.89±0.46	26.63±1.30	1.21±0.03	
***5mg/kg E4+1.6mg/kg P4+136ng/kg E2***		11.93±0.36	22.04±0.66	1.19±0.03	
***5mg/kg E4+16mg/kg P4***		**15.94±0.29**	25.58±1.47	1.17±0.02	*
***5mg/kg E4+16mg/kg P4+136ng/kg E2***		14.27±0.70	21.55±0.68	1.16±0.03	
***5mg/kg E4+136ng/kg E2***		11.94±0.59	23.91±1.34	1.12±0.03	
***10mg/kg E4***		13.35±0.47	27.10±0.83	1.19±0.02	
***10mg/kg E4+1.6mg/kg P4***		12.83±0.66	25.57±0.99	1.25±0.01	
***10mg/kg E4+1.6mg/kg P4+136ng/kg E2***		13.59±0.50	27.91±1.18	1.23±0.02	
***10mg/kg E4+16mg/kg P4***		13.55±0.34	26.46±1.20	1.23±0.03	
***10mg/kg E4+16mg/kg P4+136ng/kg E2***		13.15±0.25	27.99±1.03	1.24±0.01	
***10mg/kg E4+136ng/kg E2***		12.47±0.52	25.69±0.93	1.21±0.03	
**Treatment**					
***Sham***		12.04±0.52	26.96±0.73	1.20±0.01	
***Vehicle***		13.43±0.35	26.63±0.71	1.17±0.02	
***5mg/kg E4***		14.14±0.62	28.21±1.23	1.28±0.01	
***5mg/kg E4+1.6mg/kg P4***		14.08±0.65	27.16±0.66	1.23±0.02	
***5mg/kg E4+1.6mg/kg P4+136ng/kg E2***		13.66±0.43	25.67±0.88	1.29±0.01	
***5mg/kg E4+16mg/kg P4***		13.51±0.41	27.11±1.18	1.26±0.03	
***5mg/kg E4+16mg/kg P4+136ng/kg E2***		15.03±0.49	28.13±1.37	1.22±0.03	
***5mg/kg E4+136ng/kg E2***		13.93±0.37	24.43±2.07	1.15±0.05	
***10mg/kg E4***		14.25±0.59	30.82±0.54	**1.34±0.01**	**
***10mg/kg E4+1.6mg/kg P4***		13.83±0.66	24.97±0.89	1.20±0.02	
***10mg/kg E4+1.6mg/kg P4+136ng/kg E2***		13.44±0.47	25.48±1.22	1.25±0.02	
***10mg/kg E4+16mg/kg P4***		13.93±0.39	25.98±0.74	1.32±0.01	
***10mg/kg E4+16mg/kg P4+136ng/kg E2***		14.17±0.51	32.41±0.96	1.26±0.02	
***10mg/kg E4+136ng/kg E2***		15.04±0.42	28.64±2.76	1.22±0.04	

Significant differences were observed:

* Body weight at P7- 5mg/kg/d E4+16mg/kg/d P4 group *vs*. sham, vehicle and 5mg/kg/d E4+136ng/kg/d E2 in pretreated groups;

** Brain weight- 10mg/kg/d E4 *vs*. vehicle in treated groups.

### Hematoxylin-eosin staining and intact cell counting

**Figure 3:**
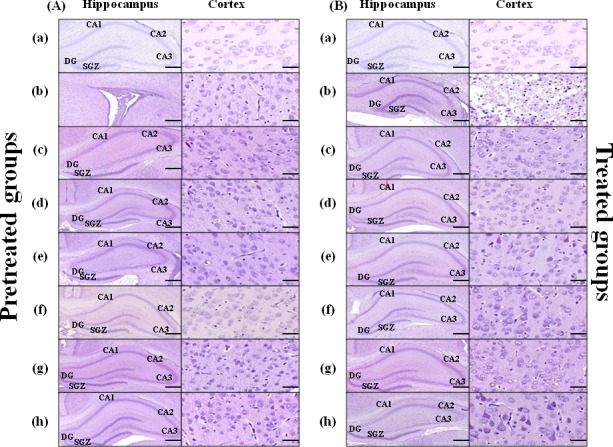
Hematoxylin-eosin staining of the brain coronal sections from rat pups pretreated/treated by E4 alone or in combination with P4 and/or E2 Brain coronal sections (Scale bar: 2mm) with hippocampus region (Scale bar: 500μm), and Cortex (Scale bar: 100μm) from pretreated **A.** and treated **B.** study groups are shown: sham (a), vehicle (b), 5mg/kg/dayE4 (c), 5mg/kg/day E4+16mg/kg/day P4+136ng/kg/day E2 (d), 5mg/kg/day E4+136ng/kg/day E2 (e), 10mg/kg/day E4 (f), 10mg/kg/day E4+16mg/kg/day P4 (g), 10mg/ kg/day E4+136ng/kg/day E2 (h).

Coronal sections from rat pups brains pretreated/treated by the vehicle showed injury of the hippocampus at the left carotid artery occlusion side which was extended to the cortex (Figure 3A(b), 3B(b)). As demonstrated in Table 2 in the hippocampal dentate gyrus (DG) region in pretreated groups the sham group showed significantly higher intact cell counting compared to the animals pretreated either with the vehicle or by 5mg/kg/d E4 and E2 with/without 16mg/kg/d P4 (Figure 3A(c), (d), respectively) or by 10mg/kg/d E4 with E2 and 16mg/kg/d P4 (Supp. Figure 1 (A)). Significantly higher number of intact cells was observed in animals pretreated by 5mg/kg/d E4 (Figure 3A(c)) and 10mg/kg/d E4 (Figure 3A (f)) alone or in combination with 1.6mg/kg/d P4 and/or E2, also in animals pretreated with 10 mg/kg/d and 16 mg/kg/d P4 compared to the vehicle group (Table 2). Among pretreated groups, significant differences were defined between animals pretreated by 10mg/kg/d E4 alone or in combination with E2 and/or any concentration of P4, and the sham group (Table 2).

**Table 2:** Intact cell counting/per visual field in the hippocampus and cortex in hematoxylin-eosin stained sections

Groups	DG	SGZ	CA1	CA2/CA3	Cortex
**Pretreatment**					
***Sham***	202.70±18.28*	**89.80±8.57§§§**	55.40±3.34	44.70±2.38	47.20±3.90
***Vehicle***	76.30±10.23 **	**33.20±5.22 #**	48.30±6.65	33.80±3.93	33.30±3.24
***5mg/kg E4***	183.40±11.96	72.10±5.93	48.60±2.57	51.80±3.56	39.80±2.74
***5mg/kg E4+1.6mg/kg P4***	168.80±17.52	62.50±6.97	54.00±3.11	45.30±2.89	36.50±3.19
***5mg/kg E4+1.6mg/kg P4+136ng/kg E2***	168.80±4.71	67.7±3.32	53.60±3.47	40.80±3.06	43.60±4.02
***5mg/kg E4+16mg/kg P4***	148.60±12.97	68.60±5.15	51.70±5.69	54.60±7.00	41.90±4.51
***5mg/kg E4+16mg/kg P4+136ng/kg E2***	167.40±13.96	73.90±7.96	45.00±6.07	44.40±6.59	40.20±3.60
***5mg/kg E4+136ng/kg E2***	111.40±6.69	42.90±3.09	56.20±3.76	38.50±2.85	36.50±3.37
***10mg/kg E4***	227.30±14.18 ***	86.10±5.84	64.90±3.63	42.40±2.49	39.60±2.86
***10mg/kg E4+1.6mg/kg P4***	166.90±11.97	64.30±5.26	58.30±3.53	45.30±2.92	45.40±5.70
***10mg/kg E4+1.6mg/kg P4+136ng/kg E2***	123.4±7.25	54.20±4.84	65.90±4.91	46.10±7.72	41.90±2.65
***10mg/kg E4+16mg/kg P4***	174.60±12.38	73.50±6.97	58.40±4.74	46.30±4.57	38.40±5.45
***10mg/kg E4+16mg/kg P4+136ng/kg E2***	115.70±6.51	52.70±2.49	45.00±3.63	46.30±5.02	36.90±3.485
***10mg/kg E4+136ng/kg E2***	131.70±8.94	56.30±3.02	57.70±2.46	43.30±1.89	39.50±4.37
**Treatment**					
***Sham***	202.70±18.28	**89.80±8.57 ##**	55.40±3.34	**44.70±32.38 †**	47.20±3.90
***Vehicle***	**74.00±10.61§**	39.10±6.79	41.80±6.86	24.30±4.15	**20.30±2.33 ††**
***5mg/kg E4***	142.90±5.63	60.40±3.55	56.30±3.45	32.90±31.62	32.70±2.64
***5mg/kg E4+1.6mg/kg P4***	138.80±14.01	45.20±4.20	53.20±5.67	34.60±33.21	42.30±3.72
***5mg/kg E4+1.6mg/kg P4+136ng/kg E2***	160.00±7.29	61.10±3.94	56.50±2.79	39.50±31.46	41.40±4.79
***5mg/kg E4+16mg/kg P4***	175.50±7.84	61.30±3.08	55.10±3.85	42.5±31.87	50.90±4.87
***5mg/kg E4+16mg/kg P4+136ng/kg E2***	164.00±12.45	58.4±4.92	57.50±4.17	35.6±33.45	37.00±4.17
***5mg/kg E4+136ng/kg E2***	128.50±12.15	54.10±4.37	54.20±4.95	38.00±34.13	46.00±4.51
***10mg/kg E4***	150.20±9.43	57.30±5.19	51.20±2.82	33.50±0.98	46.60±1.83
***10mg/kg E4+1.6mg/kg P4***	132.20±13.68	41.90±2.88	47.10±6.64	37.60±33.36	31.30±3.83
***10mg/kg E4+1.6mg/kg P4+136ng/kg E2***	156.00±6.11	56.70±2.45	61.50±2.88	39.80±31.30	38.80±4.81
***10mg/kg E4+16mg/kg P4***	168.40±4.38	65.40±3.09	57.50±1.73	40.40±31.83	48.10±4.02
***10mg/kg E4+16mg/kg P4+136ng/kg E2***	150.00±9.92	54.40±4.01	50.90±4.59	32.60±32.82	39.10±3.30
***10mg/kg E4+136ng/kg E2***	**103.40±13.47 §§**	41.50±5.55	48.40±6.86	28.90±35.34	39.10±3.45

Significant differences were observed:

**in the DG region**

***pretreated groups:***

* sham *vs*. 5mg/kg/d E4+136ng/kg/d E2, 5mg/kg/d E4+16mg/kg/d P4+136ng/kg/d E2, 10mg/kg/d+16mg/kg/d P4+136ng/kg/d E2;

**vehicle *vs*. sham, 5mg/kg/day E4, 5mg/kg/day E4+1.6mg/kg/d P4, 5mg/kg/day E4+1.6mg/kg/d P4+136ng/kg/d E2, 10mg/kg/d E4, 10mg/kg/day E4+1.6mg/kg/d P4, 10mg/kg/day E4+16mg/kg/d P4;

***10mg/kg/d E4 vs. vehicle, sham, 10mg/kg/d E4+1.6mg/kg/d P4+136ng/kg/d E2, 10mg/kg/d E4+16mg/kg/d P4+136ng/kg/d E2, 10mg/kg/d E4+136ng/kg/d E2;

***treated groups:***

§vehicle *vs*. sham, 5mg/kg/d E4+1.6mg/kg/d P4+136ng/kg/d E2, 5mg/kg/d E4+16mg/kg/d P4, 5mg/kg/d E4+16mg/kg/d P4+136ng/kg/d E2, 10mg/kg/d E4+1.6mg/kg/d P4+136ng/kg/d E2, 10 mg/kg/d E4, 10mg/kg/d E4+16mg/kg/d P4, 10mg/kg/d E4+16mg/kg/d P4+136ng/kg/d E2;

§§10mg/kg/d E4+136ng/kg/d E2 vs. sham group in treated groups;

**in the SGZ**

***pretreated groups:***

§§§sham *vs*. 5mg/kg/d E4+136ng/kg/d E2;

#vehicle *vs*. sham, 5mg/kg/d E4, 5mg/kg/d+16mg/kg/d P4+136ng/kg/d E2, 10mg/kg/d E4, 10mg/kg/d E4+16mg/kg/d P4;

***treated groups:***

## sham *vs*. vehicle, 5mg/kg/d+136ng/kg/d E2, 5mg/kg/d+1.6mg+kg+d P4, 10mg/kg/d E4+1.6mg/kg/d P4, 10mg/kg/d E4+1.6mg/kg/d P4+136ng/kg/d E2, 10mg/kg/d E4+16mg/kg/d P4+136ng/kg/d E2, 10mg/kg/d E4+136ng/kg/d E2;

**in the CA2/CA3 region**

***treated groups:***

†sham *vs*. vehicle in treated groups;

in the Cortex-

***treated groups:***

††vehicle *vs*. sham, 5mg/kg/d+16mg/kg/d P4, 10mg/kg/d, 10mg/kg/d+16mg/kg/d P4;

In treated groups (Figure 3B), in the hippocampus DG region the number of intact cells was significantly decreased in animals from the vehicle group compared to the sham group, also in animals treated by combination of different doses of E4 either with any dose of P4 and/or E2. Intact cell number was significantly downregulated in animals combinedly treated by 10mg/kg/d E4 and E2 (Figure 3B (h)) compared to the sham group (Table 2).

In pretreated groups, in the subgranular region (SGZ) of hippocampus, the sham group had significantly higher intact cell counting than animals pretreated by 5mg/kg/d E4 with E2 (Figure 3A (h)), whereas the number of intact cells was significantly downregulated in animals from the vehicle group compared to sham group and the groups pretreated by different doses of E4 alone or combined with 16mg/kg/d P4. The same pattern of significant difference was observed in animals pretreated by 5mg/kg/d E4 in combination with 16mg/kg/d P4 plus E2 and the vehicle group (Table 2).

In treated groups, in the SGZ region significant differences were observed between the vehicle group and the animals treated by different doses of E4 with 1.6mg/kg/d P4 or E2, also the animals treated by 10mg/kg/d E4 with E2 and different doses of P4 (Supx2p. Figure 1 (B)), (Table 2). In the CA2/CA3 region among treated groups the intact cell counting was significantly different only among animals from sham and the vehicle groups, whereas in the cortex significantly higher number of intact cells was detected in sham group along with groups treated either by different doses of E4 in combination with16mg/kg/d P4 or 10mg/kg/d E4 alone (Figure 3B) and the vehicle group (Table 2).

### MAP-2 staining

**Figure 4:**
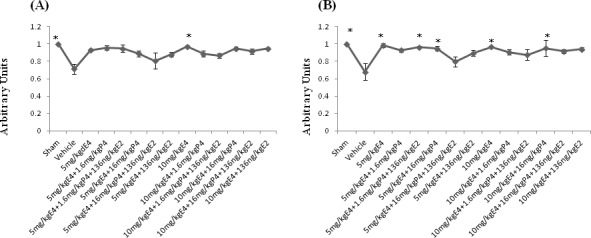
MAP-2 staining of brain coronal sections from rat pups pretreated/treated by E4 alone or in combination with P4 and/or E2 For evaluation of gray matter loss MAP2 staining was performed. **A.** Among pretreated groups the MAP2-positively stained area ratio was significantly upregulated in animals pretreated by 10mg/kg/day E4 alone than in the vehicles as well as in animals from sham group. **B.** After treatment with different combinations of steroids, MAP-2 positive area ratio was significantly higher along with the sham group in groups treated by 5mg/kg/day or 10mg/kg/day E4 alone or in combination with 16mg/kg/day P4 compared to the vehicle group. The similar pattern was observed in animals combinedly treated by 5mg/kg/day E4 with 1.6mg/kg/day P4 and 136ng/kg/day E2. 10 samples from each group were analyzed. The ratio of the MAP2 positive area in sham operated animals was considered as 1.0 by default. All measurements are expressed as mean±SEM.*p ≤ 0.05.

For evaluation of gray matter loss MAP2 staining was used. In sections from the vehicle pretreated/treated animals was observed an existence of MAP2 negatively stained areas in the hippocampus and the cortex at the left, damaged side (Supp. Figure 2 (A), (B)). Calculations of MAP-2 positive area ratios showed that after pretreatment with different combinations of steroids the ratio of MAP2-positively stained area was significantly upregulated in animals pretreated by 10mg/kg/d E4 alone than in the vehicles (Figure 4A) as well as in animals from sham group. Figure 4B shows that after treatment with different combinations of steroids, MAP-2 positive area ratio was significantly higher along with the sham group in groups treated by E4 alone or in combination with 16mg/kg/d P4 compared to the vehicle group. The similar pattern was detected in animals combinedly treated by 5mg/kg/d E4 with 1.6mg/kg/d P4 and E2. Treatment with 5mg/kg/d E4 alone or combined either with 1.6mg/kg/P4 and E2 or 16mg/kg/d P4 restored the MAP-2 positive area ratio almost to the sham level.

### Doublecortin and vascular endothelial growth factor double-immunofluorostaining

**Figure 5:**
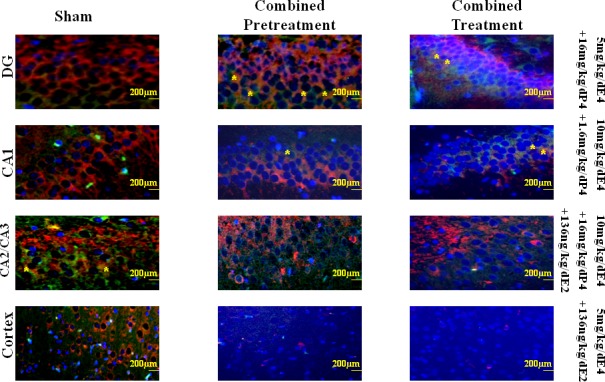
Representative views of double-labeled immunofluorescent sections from different regions of the hippocampus and the cortex from groups pretreated/treated with E4 alone or in combination with P4 and/or E2 To determine the localization and expression of DCX and VEGF in different regions of the hippocampus and the cortex the double immunofluorescent staining was performed. Red cells denote the DCX positively stained cells, whereas green cells denote the VEGF positively stained cells. Asterisks indicate co-localization of DCX and VEGF positively stained cells. Scale bar: 200 μm.

Markers for neuro- and vasculogenesis, doublecortine (DCX) and vascular endothelial growth factor (VEGF), respectively, were studied in brain sections (Figure 5). Combined pretreatment with E4 and other steroids resulted in significant upregulation of DCX expression in the DG region (the hippocampus) in 5mg/kg/d E4 and sham groups compared to the vehicles (Table 3), whereas the expression of VEGF was significantly upregulated in animals pretreated by 10 mg/kg /d E4 along with 1.6mg/kg/d P4 and E2 and the sham group compared to the vehicles (Table 3). Furthermore, in the CA1 region significant difference of the DCX expression levels was detected between sham and the vehicle groups, whereas angiogenesis was significantly increased in animals combinedly pretreated by 5mg/kg/d E4 and 16mg/kg/d P4 and the sham group compared to the vehicle group (Table 3). In the CA2/CA3 region expressions of DCX and VEGF were significantly different between sham and the vehicle groups. In the cortex neuro-and angiogenesis were significantly different between the animals from sham group and the animals pretreated by 5mg/kg/d E4 and 16mg/kg/d P4 compared to animals from the vehicle group (Table 3).

**Table 3:** Percentage of the DCX and VEGF-positively stained cells in combinedly pretreated/treated groups

Groups	DG	CA1	CA2/CA3	Cortex	DG	CA1	CA2/CA3	Cortex
	DCX	DCX	DCX	DCX	VEGF	VEGF	VEGF	VEGF
**Pretreatment**								
***Sham***	59.73±3.44	52.34±30.99	51.25±2.42	63.34±2.17	52.38±1.58	51.87±3.68	55.79±2.92	58.29±2.98
***Vehicle***	30.90±3.97 *	**16.78±32.36** **###**	**15.97±2.35** **†**	29.48±1.98 *#	31.25±3.70 **	**20.76±2.71** **§**	**20.05±3.01** **††**	28.47±2.13 **#
***5mg/kg E4***	60.29±5.66	45.97±34.40	32.38±5.03 *§	51.47±3.98	42.81±2.68	49.99±3.94	43.80±3.68	46.00±2.55
***5mg/kg E4 +1.6mg/kg P4***	45.16±3.26	42.67±35.07	34.03±5.62	51.89±5.26	38.60±3.32	44.96±5.04	42.96±3.70	40.31±4.95
***5mg/kg E4+1.6mg/kg P4+136ng/kg E2***	58.55±5.37	37.08±32.67	22.68±2.81	53.98±3.81	47.71±4.36	36.17±3.17	31.14±2.87	39.23±2.69
***5mg/kg E4 +16mg/kg P4***	56.23±4.02	51.34±34.00	49.02±9.09	71.46±4.89	44.53±2.50	59.56±3.26	51.57±6.77	60.22±4.09
***5mg/kg E4+16mg/kg P4+136ng/kg E2***	53.40±3.56	32.41±39.50	27.43±9.36	53.06±3.10	46.67±2.59	42.49±8.47	41.07±7.66	49.67±4.23
***5mg/kg E4 +136ng/kg E2***	33.49±4.53	42.61±35.66	36.19±3.25	52.05±3.92	46.84±2.56	50.07±4.74	46.74±3.87	43.20±4.02
***10mg/kg E4***	50.43±4.56	39.96±33.60	26.58±2.8	56.12±6.10	51.44±3.29	49.21±3.49	38.31±4.33	52.90±5.81
***10mg/kg E4 +1.6mg/kg P4***	45.02±5.92	31.59±36.83	18.11±3.46	44.88±7.95	42.77±3.97	39.57±6.49	39.64±6.07	46.93±6.38
***10mg/kgE4+1.6mg/kg P4+136ng/kg E2***	53.25±3.17	38.75±35.03	36.12±4.79	56.97±7.53	55.34±2.35	34.83±3.38	42.80±5.43	52.93±5.54
***10mg/kg E4 +16mg/kg P4***	44.50±2.96	32.18±35.74	27.73±6.12	49.22±4.72	47.05±3.30	32.57±4.74	34.51±7.05	38.10±4.57
***10mg/kg E4+16mg/kg P4+136ng/kg E2***	45.86±3.28	30.73±34.99	22.41±4.47	53.04±5.04	43.87±3.22	37.30±3.13	36.25±6.02	38.03±3.25
***10mg/kg E4 +136ng/kg E2***	39.25±1.63	41.02±34.96	46.71±3.76	52.02±3.34	48.40±2.66	49.99±3.20	51.74±5.20	45.40±3.63
**Treatment**								
***Sham***	**59.73±3.44** **#**	52.43±0.99	51.25±2.42	63.34±2.17	52.38±1.58	51.87±3.68	55.79±2.92	58.29±2.98
***Vehicle***	30.25±4.90 ***	**23.17±4.43** **§§**	**16.96±3.67** **†††**	31.01±3.90 *##	**29.87±2.88** **##**	**21.95±2.48** **§§§**	22.93±2.37 *§§	27.85±2.80 *†
***5mg/kg E4***	55.72±2.91	36.63±4.00	15.09±2.12	61.28±6.10	54.05±3.51	36.54±3.15	34.12±3.54	40.72±4.85
***5mg/kgE4*** ***+1.6mg/kg P4***	37.27±3.44	34.00±5.20	24.45±5.83	52.05±6.93	43.07±3.66	36.96±7.55	40.78±9.06	44.91±5.89
***5mg/kg E4+1.6mg/kg P4+136ng/kg E2***	34.85±2.10	36.95±3.00	30.95±3.64	53.75±4.25	41.34±2.37	35.08±3.94	33.83±5.17	45.44±3.91
***5mg/kg E4 +16mg/kg P4***	53.06±2.55	43.67±3.16	46.37±3.61	47.08±5.96	54.97±3.07	49.78±2.58	52.41±4.25	45.84±5.02
***5mg/kg E4+16mg/kg P4+136ng/kg E2***	41.97±2.02	43.27±2.84	38.16±3.91	39.14±3.92	42.71±3.02	44.08±4.12	35.17±3.88	34.58±3.06
***5mg/kg E4 +136ng/kg E2***	41.69±2.75	54.47±4.17	43.03±5.13	53.37±3.72	40.10±4.40	50.32±3.00	43.75±3.83	36.50±3.17
***10mg/kg E4***	43.67±2.26	41.08±2.47	36.18±3.70	53.88±3.90	44.65±3.38	46.16±2.96	49.14±3.84	46.66±3.90
***10mg/kg E4 +1.6mg/kg P4***	49.64±4.35	45.286±4.01	52.53±5.02	44.61±5.31	43.31±3.97	51.91±1.57	49.58±4.25	41.96±6.01
***10mg/kgE4+1.6mg/kg P4+136ng/kg E2***	37.73±3.38	44.74±7.93	31.47±6.98	47.19±6.10	46.97±3.91	39.40±3.79	40.78±4.16	34.81±3.12
***10mg/kg E4 +16mg/kg P4***	41.10±4.00	48.83±2.69	46.83±3.53	62.66±3.74	46.85±3.19	50.72±3.83	52.68±3.94	48.70±3.76
***10mg/kg E4+16mg/kg P4+136ng/kg E2***	33.53±1.70	30.11±2.24	27.84±4.31	42.81±2.93	45.08±1.75	40.39±3.51	39.24±4.96	37.98±3.76
***10mg/kg E4 +136ng/kg E2***	39.89±3.20	38.39±6.74	42.00±5.22	46.20±3.74	47.03±3.82	44.97±8.41	40.61±6.51	39.07±3.32

Significant differences were observed:

**in the DG region**

***pretreated groups:***

DCX-stained cells -*vehicle *vs*. sham, 5mg/kg/d E4, VEGF-stained cells-**vehicle *vs*. sham, 5mg/kg/d E4+16mg/kg/d P4;

***treated groups:***

DCX stained cells-*** vehicle *vs*. sham, 5mg/kg/d E4, 5mg/kg/d E4+16mg/kg/d P4, # sham *vs*. 5mg/kg/d E4+1.6mg/kg/d P4, 5mg/kg/d E4+1.6mg/kg/d P4+136ng/kg/d E2, 10mg/kg/d E4+1.6mg/kg/d P4+136ng/kg/d E2, 10mg/kg/d E4+16mg/kg/d P4+136ng/kg/d E2;

VEGF stained cells-## vehicle *vs*. sham, 5mg/kg/d E4, 5mg/kg/d E4+16mg/kg/d P4;

**in the CA1 region**

***pretreated groups:***

DCX-stained cells-### vehicle *vs*. sham, VEGF-stained cells-§ vehicle *vs*. sham, 5mg/kg/d+16mg/kg/d P4;

***treated groups:***

DCX-stained cells-§§vehicle *vs*. sham, 5mg/kg/d E4+136ng/kg/d E2, VEGF-stained cells-§§§vehicle *vs*. sham, 10mg/kg/d E4+1.6 mg/kg/d P4;

**in the CA2/CA3 region**

***pretreated groups:*** DCX-stained cells-† vehicle vs. sham, VEGF stained cells- †† vehicle vs. sham;

***treated groups:*** DCX-stained cells-†††vehicle vs. sham, 10mg/kg/d+1.6mg+kg+d P4, *§ 5mg/kg/d E4 *vs*. sham, 5mg/kg/d E4+16mg/kg /d P4, VEGF stained cells-**§ vehicle vs. sham,

**in the Cortex**

***pretreated groups:***

DCX-stained cells-*#vehicle *vs*. sham, 5mg/kg/d E4+16mg/kg/d P4, VEGF-stained cells-**# vehicle *vs*. sham, 5mg/kg/d E4+16mg/kg/d P4;

***treated groups:*** DCX-stained cells- *## vehicle *vs*. sham, VEGF-stained cells- *†vehicle *vs*. sham.

Treatment of animals after HI insult with E4 alone or combined with other steroids resulted in significant upregulation of DCX expression in the hippocampus in animals treated by 5mg/kg/d E4 alone or with 16mg/kg/d P4 compared to the vehicle group (Figure 5). Also, sham group showed significantly higher number of DCX positively stained cells than the groups combinedly treated either by 5mg/kg/d with 1.6mg/kg/d P4 or 10mg/kg/d E4 with 16mg/kg/d P4 and E2 as well as groups treated by E4 in combination with 1.6mg/kg/d P4 and E2 (Table 3). In the same region the VEGF positively stained cells percentage was significantly increased in the sham operated animals and in groups treated by 5mg/kg/d E4 alone or in combination with 16mg/kg/d P4 than in the vehicle group. Furthermore, in the CA1 region neurogenesis was significantly upregulated in sham group and in animals treated by 5mg/kg/d E4 along with E2 than in the vehicles, whereas angiogenesis was significantly upregulated in sham group and in animals treated by 10mg/kg/d E4 with 1.6mg/kg/d P4 compared to the vehicles (Table 3). In the CA2/CA3 region significant differences in DCX expression was detected between sham group, the animals treated by 10mg/kg/d E4 with 1.6mg/kg/d P4 and the vehicles as well as between sham group, the animals treated by 5mg/kg/d E4 with 16mg/kg/d P4 and the group treated by 5mg/kg/d E4 alone (Table 3). In the same region VEGF was significantly expressed in sham group than in the vehicles. In the cortex neuro- and angiogenesis were significantly upregulated in sham group compared to the vehicle group. In general, combination of E4 with E2 resulted in low DCX and VEGF expression levels in the cortex.

### Blood serum glial fibrillary acidic protein (GFAP) expression

Glial fibrillary acidic protein (GFAP) expression, as brain damage marker, was evaluated in blood sera by using ELISA. As shown in Table 4, combined pretreatment by E4 with P4 and/or E2 resulted in significant difference of GFAP concentration levels between the sham-operated group and the animals pretreated by the vehicle or by 10mg/kg/d E4 in combination either with E2 or 1.6mg/kg/d P4. Animals pretreated by 5mg/kg/d E4 with E2 and P4 had significantly lower level of GFAP protein than the vehicle group. Significant downregulation of GFAP concentration was also observed in animals pretreated either by 5mg/kg/d E4 alone or in combination with different doses of P4 and E2 or combined with 16mg/kg/d P4 and in sham group than in animals pretreated by 5mg/kg/d E4 and E2 (Table 4). The same pattern of GFAP concentration differences were observed between groups pretreated by 10mg/kg/d E4 alone or in combination with different doses of P4 and E2 or with 16mg/kg/d P4 than in animals pretreated by 10mg/kg/d E4 and E2 (Table 4).

**Table 4:** Glial fibrillary acidic protein (GFAP) expression in blood serum (pg/ml) of the combinedly pretreated/treated rat pups

Groups	Combined Pretreatment		Combined Treatment	
	pg/ml	N of samples	pg/ml	N of samples
***Sham***	2393.40±1454.429*	8	**2393.40±1454.43##**	8
***Vehicle***	23915.91±3158.84**	10	28901.155±4480.30	11
***5mg/kg E4***	6220.49±1763.17	11	6380.10±4062.591	10
***5mg/kg E4+1.6mg/kg P4***	12548.31±2280.50	10	8146.34±3596.07	10
***5mg/kg E4+1.6mg/kg P4+136ng/kg E2***	1011.42±55.32	13	19226.69±2559.70	10
***5mg/kg E4+16mg/kg P4***	5113.67±1733.57	10	25919.72±4487.50	10
***5mg/kg E4+16mg/kg P4+136ng/kg E2***	737.01±69.82	11	17476.73±2643.53	10
***5mg/kg E4+136ng/kg E2***	28442.46±3457.11***	11	32354.42±5946.66	10
***10mg/kg E4***	12413.45±2243.05	12	**10806.52±1915.19###**	10
***10mg/kg E4+1.6mg/kg P4***	27225.88±8442.88	7	18796.20±4279.45	10
***10mg/kg E4+1.6mg/kg P4+136ng/kg E2***	9672.46±2461.11	12	20470.58±1468.47	14
***10mg/kg E4+16mg/kg P4***	12037.18±3726.66	12	15974.26±2111.42	11
***10mg/kg E4+16mg/kg P4+136ng/kg E2***	11202.39±2765.16	11	22202.18±2624.61	11
***10mg/kg E4+136ng/kg E2***	**32898.22±3437.25#**	11	26660.81±4870.81	10

Significant differences were observed:

**in pretreated groups:**

*sham *vs*. vehicle, 10mg/kg/d E4+136ng/kg/d E2, 10mg/kg/d E4+1.6mg/kg/d P4,

** vehicle *vs*. 5mg/kg/d E4+1.6mg/kg/d P4+136ng/kg/d E2, 5mg/kg/d E4+16mg/kg/d P4+136ng/kg/d E2,

*** 5mg/kg/d E4+136ng/kg/d E2 *vs*. sham, 5mg/kg/d E4, 5mg/kg/d E4+1.6mg/kg/d P4+136ng/kg/d E2, 5mg/kg/d E4+16mg/kg/d P4, 5mg/kg/d E4+16mg/kg/d P4+136ng/kg/d E2,

#10mg/kg/d E4+136ng/kg/d *vs*. sham, 10mg/kg/d E4, 10mg/kg/d E4+1.6mg/kg/d P4+136ng/kg/d E2, 10mg/kg/d E4+16mg/kg/d P4, 10mg/kg/d E4+16mg/kg/d P4+136ng/kg/d E2 ;

**in treated groups:**

##sham *vs*. vehicle, 5mg/kg/d E4+136ng/kg/d E2, 10mg/kg/d E4+136ng/kg/d E2,

###10mg/kg/d E4 *vs*. 10mg/kg/d E4+136ng/kg/d E2.

Treatment by E4 with P4 and/or E2 resulted in significant decrease of GFAP protein concentration in 10 mg/kg/d E4 and the sham groups than in animals treated by combination of 10mg/kg/d E4 and E2. Also, the sham-operated group had significantly lower concentration of GFAP than the vehicle group and animals treated by E4 and E2. In both pretreated and treated groups the combination of E4 and E2 showed significantly higher levels of GFAP, suggesting a negative cooperativity of these steroids upon cell survival.

## DISCUSSION

Our results evaluate the efficacy of E4 administration alone or in combination with P4 and/or E2 for neuroprotection in a 7-day-old rat pups model of hypoxic-ischemic brain damage. Neurogenesis in humans starts around day 33 of the embryonal development and by 8-9 wks of gestation the cortical plate is usually formed [53]. Both estrogen receptors: α (ERα) and β (ERβ), are expressed in the human cortex and hippocampus during neurodevelopment. ERα, detected by 9 weeks of gestation, plausibly has importance for the early neurodevelopment, whereas ERβ might have importance for later processes, such is corticogenesis [54]. In rats, estrogen receptors present in developing brain as well as in adults brains mainly in the hippocampus [55, 56] with a significant binding properties at postnatal day 4 (P4) which declines to adult levels by P15 [57, 58]. The expression of ERα mRNA in the neonatal cortex and/or olfactory bulb and the cerebellum supports the notion that ERα might be connected to cellular differentiation as well as to sexual differentiation of the brain during neurodevelopment [59, 60]. It might, in addition, have a role in estradiol-dependent protection against delayed cell death [61], and as it was recently shown, in genetically modified animals with middle cerebral artery occlusion (MCAO) the ERα might have importance in neuroprotection supported by estrogens [62]. Estrogens neuroprotective properties are realized through different pathways, for example, estrogens may increase the astrocyte's possibility to absorbe glutamate and like that prevent the glutamate toxicity-mediated neuronal loss [63, 64]. Estrogens also have an importance in suppression of neuroinflammation [65], and activation ERK pathways which are necessary for the maintenance of neuritic arborisation and neuronal morphology [66]. Recent studies proved that the neuroprotective actions of estrogens also depend on their strong antioxidant specifications and positively correlate with the number of the phenolic moiety in their structure; existance of the free phenolic OH group is absolutely important for protection against oxidative stress [67]. The highest number of the free phenolic OH groups among estrogens is in E4 suggesting the strong antioxidant effects of this compound. Much research has been done to study the mitochondria as a primary target for estrogen-mediated pathways [68-72]. Furthermore, estrogens may increase aerobic glycolysis, generation ATP along with the increase of the Ca2+ load tolerance leading to the antioxidant defense [73]. As it was already demonstrated, estrogen receptor activation offers neuroprotection, in part, through transcriptional mechanisms affecting the apoptotic cascade including BCl2, caspases and Apaf-1 [31, 74-76] like that limiting the cell death. Another way for estrogen-mediated neuroprotection might be connected to the direct activation of pathways involving MAP-kinase by ER [77].

On the other hand, the rat forebrain expresses high levels of progesterone receptors (PR) as early as E17-E18 in regions with important cognitive, motor and visual functions, pointing out the importance of the hippocampus in establishment of early cortical circuitry. P4 neuroprotective effects involve the activation of a number of pathways that influence inflammatory and oxidative mechanisms, and the repair processes that follow in response to injury. In the adult rat brain, the upregulation of nitric oxide synthase-2 (NOS-2), involved in production of nitric oxide free radicals, and pro-inflammatory IL-1β after ischemic events caused by MCAO is inhibited by progesterone treatment [78]. P4 also reduces the proliferation of reactive astrocytes, and inflammatory prostaglandin synthesis further leading to the reduction of edema and the blood-brain barrier leakage in adults after traumatic brain injury [16, 79]. Also, it influences post-ischemic synaptogenesis in the specific region of the hippocampus such is CA1 [80]. P4 is linked to activation of ERK, MAPK and PI3K/Akt pathways that are neuroprotective against glutamate-induced cell death, having inhibitory effect on neuronal apoptosis leading to the alleviation of HIE [26, 41]. P4 may induce a neuroprotective effect by upregulating expression of brain-derived neurotrophic factor (BDNF) which is a nerve growth factor that promotes neuron survival and formation of synapses [26, 81]. It was demonstrated that P4 has a promyelinating effect by promoting increase of myelin basic protein expression (MBP) [82]. Furthermore, it might upregulate the well-known inhibitory transmitter GABAa and may reduce the apoptosis by downregulation of NFκB [83-85]. Taken together, P4 along with E2 plays a critical role in neuronal developmental processes not only in prenatal period but in adulthood as well [86, 87].

Recent studies already demonstrated possible link between E4, E2 and P4 in the CNS. 5mg/kg/day E4 increased allopregnanolone (3-hydroxy-5-pregnan-20-one (AP), a well-known metabolite of P4, rates in different brain regions and in the serum of ovariectomized (OVX) animals. However, the presence of E2 abolished these effects mediated by E4 [34]. Our previous studies demonstrated that the concomitant exposure to E2 and E4 *in vitro* and *in vivo* resulted in a partially antagonized effect of E4 on the proliferation induced by E2 on HBE cells and on mammary gland growth [88]. These observations are in concert with our present results connected to the concomitant use of E4 and E2: neither *in vitro* nor *in vivo* combinations of E4 and E2 (except the combination of the highest dose of E4 with E2 *in vitro*) showed any significant positive result compared to the vehicle group suggesting that E4 and E2 possibly antagonize each other effects.

According to some studies, P4 might prevent estradiol-induced dendritic spine formation in cultured hippocampal cells by antagonizing the effect of E2 on hippocampal spine density or even contribute to the loss of hippocampal spines and spine synapses noted across the estrous cycle, though during the first 6 h of P4 use, it increases the hippocampal dendritic spine density [89-91]. In our present studies combined use of E4, P4 and E2 *in vitro* showed significant downregulation of LDH activity and the higher cell survival rate than the combination of E4 and E2 or even the single use of E4. These results were similar to those obtained upon the combined use of E4 and P4 suggesting that combined use of E4 and P4 potentiates each other's effects and somehow neutralizes the antagonizing effect of E4 on E2 when 3 compounds are used together. *In vivo* studies showed that some combinations of E4 with P4 and or E2 might affect the body temperature, and the combination of E4 with P4 may affect positively the body weight, but animals pretreated by combination of E4 with P4 and E2 have significantly higher brain/body ratio suggesting that combined use of E4, P4 and E2 may result in disproportional development. Only the single use of E4 after HI insult had significant effect on brain weight without affecting the brain/body ratio. E4 alone and some combinations of E4, P4 and/or E2 had positive effects on intact cells number in different hippocampal regions and the cortex, though in pretreated groups the number of intact cells was significantly upregulated in the DG region when E4 was used alone rather than combined with P4 and/or E2.

The early gray matter was significantly preserved in pretreated groups with single use of E4, whereas in combinedly treated groups the MAP-2 staining was significantly upregulated when E4 was used alone or combined with P4 and/or E2. As it was shown from our previous study [33], E4 positively affected the expression of a marker of neurogenesis (DCX), suggesting upregulation of neurogenesis, possibly through estrogen receptors. Our current study is in concert with the previous one and, moreover, it shows that combined use of E4 with P4 also affects neurogenesis suggesting that neurogenesis might be upregulated not only through estrogen receptors but through progesterone receptors as well, though involvement of some other mechanisms is also plausible. As it was shown recently, VEGF, an angiogenic protein, has impressive neurotrophic and neuroprotective effects by stimulating neurogenesis *in vitro* and *in vivo* in the subventricular zone (SVZ) and the subgranular zone (SGZ) of the brain [92] by promoting proliferation of cortical neuron precursors through regulation of E2F expression (the family of transcription factors, a key regulator of the cell cycle machinery) [93]. On the other hand, there is an important correlation between the neuronal VEGF expression and angiogenesis in immature rat brain [92, 94]. Taken together, a mechanism linked to the neurogenesis after combined use of E4 and P4 might be also connected to elevated expression of VEGF from neuronal cells.

Some recent clinical investigations showed that the serum GFAP levels during the first week of life are increased in neonates suffering from HIE and the severity corresponds very well with MRI findings [95, 96]. Thus, GFAP might be used as marker for brain damage prediction and could help detect neonates with moderate or severe HIE. GFAP might be used as prognostic marker for evaluation of treatment efficacy as well [95, 96]. In our present study GFAP was significantly downregulated either by single dose of E4 or by its combination with P4 and E2. Evaluation of positive effects of different combinations of E4 with P4 and/or E2 in both *in vivo* models by the number of experimental benefits and the importance for each region of the brain we studied leads us to the conclusion that an important results are shown either by combination of 5mg/kg/d E4 with 16mg/kg/d P4 or by the use of 10mg/kg/d E4 alone.

In conclusion, E4 is an estrogen with SERM properties related to its dual action of agonist on the nuclear ERα and the antagonist activity on the membrane ERα, whereas E2, in contrast, activates both forms of ERα [36]. Our data further confirms the potential antagonistic activity of E4 on E2 initiated activities. We speculate that combined use of E4 does not have any priority over the isolated use of E4. One limitation of our study might be the fact that we did not compare our results with the single doses of P4 or E2, but our aim was to show that E4, a natural human estrogen solely produced in large quantities during human pregnancy [32], could be a safe and efficient candidate for treatment of early brain damage in newborns. The high selectivity of E4 and a lack of impact on coagulation and the liver [97] suggests a low risk of unexpected side-effects. Taken together, single use of E4 has enough benefits and potency to become an important safe substance for treatment of HIE.

**Figure 6:**
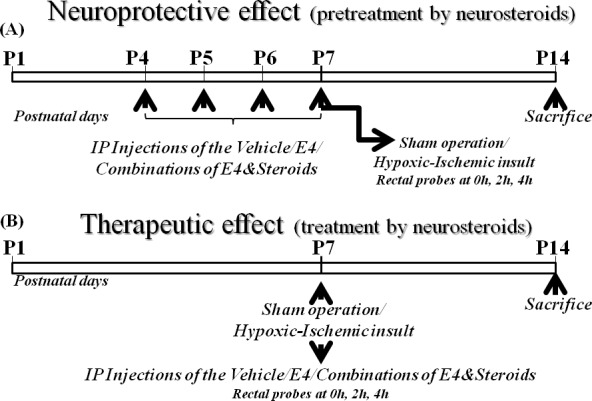
Schematic representation of *in vivo* studies **A.** E4 pretreatment (neuroprotective effect). Newborn rat pups at P4 were assigned to: sham group (neither vehicle nor E4/steroids were applied), vehicle group or groups of animals pretreated either by 5 mg/kg/d E4 and 10mg/kg/d E4 alone or in combination either with 1.6mg/kg/day P4 and/or 136ng/kg/day E2 or with 16mg/kg/day P4 and/or 136ng/kg/day E2. From P4 to P7 inclusive, the rat pups were administered ip either vehicle or E4 alone or E4 combined with other steroids or neither by vehicle nor by other compounds (sham group). At P7, animals from the vehicle and steroid groups were subjected to hypoxic-ischemic (HI) insult. The sham group passed through anesthesia and skin incision but without HI insult or injections. Rat pups were sacrificed at P14. **B.** E4 treatment (therapeutic effect). Newborn rat pups at P7 were assigned to: sham group (neither vehicle nor E4/steroids were applied), vehicle treated group or groups of animals pretreated either by 5 mg/kg/d E4 and 10mg/kg/d E4 alone or in combination either with 1.6mg/kg/day P4 and/or 136ng/kg/day E2 or with 16mg/kg/day P4 and/or 136ng/kg/day E2. At P7, animals from the vehicle and steroid groups passed through hypoxic-ischemic (HI) procedures. Upon retrieval from hypoxia chamber rat pups were administered ip either vehicle or one of combinations of E4, P4 and/or E2. The sham group went through anesthesia and skin incision but without HI insult or injections. Rat pups were sacrificed a tP14.

## MATERIALS AND METHODS

### *In vitro* studies

### Preparation of primary hippocampal neuronal cultures

We prepared primary hippocampal neuronal cultures from newborn (P0) Sprague-Dawley (SD) rat pups brains according to the recently published protocols [98, 99] which we have used in our previous study [33]. Briefly, brains were dissected to separate hippocampus region. Hippocampi were separated in dissection medium consisting of Hanks Balanced Salt Solution (HBSS), Invitrogen, Gent, Belgium) supplemented with Sodium Pyruvate (100x) (Invitrogen, Gent, Belgium), Glucose (Sigma-Aldrich, St. Louis, MO, USA), and HEPES buffer (10mM) (Sigma-Aldrich, St. Louis, MO, USA)). After washing the tissue was placed in fresh dissection medium with 2.5% trypsin solution (Invitrogen, Gent, Belgium), and further incubated in a 5% CO2, 95% air atmosphere at 37°C for 20 min. After adding DNase solution (Sigma-Aldrich, St. Louis, Mo, USA), hippocampi were incubated at room temperature for 5 min in fresh dissectin medium. Hippocampi were resuspended in 2.5 ml of plating medium consisting of Minimal Essential Medium (MEM) with Earle's salts (Invitrogen, Gent, Belgium), supplemented with 10% of fetal bovine serum (FBS) (Invitrogen, Gent, Belgium), Glucose (Invitrogen, Gent, Belgium), Sodium Pyruvate (Invitrogen, Gent, Belgium), GlutaMax-I-supplement (Invitrogen, Gent, Beligum), and Penicillin/Strepromycin (100x) (Invitrogen, Gent, Belgium). Hippocampi were dissociated and the cell viability was evaluated. The dissociated cells were plated on poly-L-lysine coated 24-well (5×10^4^Cells/well) or 96-well (5×10^3^cells/well) culture plates. Coating Poly-L-Lysine solution was prepared from Poly-L-lysine powder (Sigma-Aldrich, St. Louis, MO, USA). Cultures were incubated in a humidified 5% CO2/95% air atmosphere at 37°C in maintenance medium consisting of Neurobasal medium (Invitrogen, Gent, Belgium), containing of supplement B-27 (50x) (Invitrogen, Gent, Belgium), GlutaMax-I-supplement (Invitrogen, Gent, Belgium), and penicillin/Streptomycin (100x) (Invitrogen, Gent, Belgium). Cytosine arabinosidase (Ara-C) (Sigma-Aldrich, St. Louis, MO, USA) was added to the maintenance media 48h later after plating of the cells during 24 h. Upon changing the culture medium, the cultures were incubated for additional 3-4 days prior to use.

### Cell culture stimulation with H202, E4 alone or in combination either with E2 and/orP4

To define the concentrations of E2 (Sigma-Aldrich, St. Louis, MI, USA) and P4 (Sigma-Aldrich, St. Louis, MI, USA) with antioxidative and neuroprotective properties on primary hippocampal cell cultures prepared from newborn rat pups, at day 7 after plating cells, cultures were treated with 100μM of H202 for 30min (Merck KGaA, Darmstadt, Germany) and then with different concentrations of E2 or P4 from 1nM to 1mM for 1h. Cell cultures treated only with 100μM of H202 for 1h30 min were used as controls (data not shown). Successful concentrations (100nM of E2 and 1mM of P4) were further considered for combination studies with E4 (Pantarhei Bioscience, Zeist, The Netherlands) in primary hippocampal cell cultures. We used the same concentrations of E4 as in our previous *in vitro* studies [33]. To study an antioxidative and neuroprotective effects of E4 in combination with other steroids on primary hippocampal cell cultures at day 7 *in vitro* (DIV), they were stimulated with 100 μM of H202 for 30 min followed by treatment for 1 h either with 650μM, 3.25mM and 6.5mM E4 alone or in combination with 100nM E2 and/or 1mM P4 as followed: E4+100nM E2, E4+1mM P4, E4+100nM E2+1mM P4. Cell cultures treated only with 100μM of H202 for 1h30 min were used as controls. Supernatants were subjected to the LDH activity and the cells viability assays. The rest of the cell cultures were subjected to the cell viability assay.

### Evaluation of lactate dehydrogenase (LDH) activity

To evaluate the existance of oxidative stress and expression of LDH in primary hippocampal cell cultures stimulated by different concentrations of E4 alone or in combination with other steroids after experimental oxidative stress, a commercial LDH assay kit was used (Abcam Inc, Cambridge, MA, USA) as previously [33]. This colorimetric method for quantification of LDH activity transforms NAD to NADH. All procedures were performed in accordance to the manufacturer's protocol. Each condition was repeated 4-7 times.

### Cell viability assay

To evaluate the cell viability, the primary hippocampal cell cultures were stimulated by 100μM of H202 and followed by treatment with few doses of E4 alone or in combination with other steroids. A commercial Cell Titer 96^®^ aqueous one solution cell proliferation assay kit was used (Promega Corporation, Madison, WI, USA) as previously [33]. Each condition was repeated 3-6 times.

### *In vivo* studies

We obtained SD pregnant rats from Janvier (France). After delivery, the newborn pups were reared with their dams at 25°C. All experimental procedures were supported by the University of Liege (Belgium) Ethical Committee. The steroids used for *in vivo* studies were dissolved in absolute Ethanol (EtOH) and further diluted at a final concentration of EtOH 10% in sesame oil. An equal volume (5μl/g body weight) was injected intraperitoneally (ip) into the pups from study groups. The vehicle group animals were ip injected a sesame oil containing 10% EtOH. No injections were performed in sham group.

### Neuroprotective (pretreatment) effect of E4 alone or in combination with P4 and/or E2 (Figure 6A)

To compare the neuroprotective effect of E4 alone or E4 in combination with E2 and/or P4 the rat pups from P4 were designated different groups: sham group (*n* = 12) (without any kind of pretreatment), vehicle group (*n* = 17), 5mg/kg/d E4 (*n* = 13), 10mg/kg/d E4 (*n* = 12), 5mg/kg/d E4+1.6mg/kg/d P4 (*n* = 11), 10mg/kg/d E4+1.6mg/kg/d P4 (*n* = 11), 5mg/kg/dE4+16mg/kg/d P4 (*n* = 11), 10mg/kg/d E4+16mg/kg/d P4 (*n* = 11), 5mg/kg/d E4+136ng/kg/d E2 (*n* = 11), 10mg/kg/d E4+136ng/kg/d E2 (*n* = 11), 5mg/kg/d E4+1.6 mg/kg/d P4+136ng/kg/d E2 (*n* = 13), 10mg/kg/d E4+1.6mg/kg/d P4+136ng/kg/d E2 (*n* = 16), 5mg/kg/d E4+16mg/kg/d P4+136ng/kg/d E2 (*n* = 11), 10mg/kg/d E4+16 mg/kg/d P4+136ng/kg/d E2 (*n* = 13) groups according to the group assignment. Estetrol concentrations 5mg/kg/d and 10mg/kg/d were the most successful doses used in our previous studies [33]. A model of hypoxia-ischemia in an immature rats was used at P7 [42] with slight modifications as in our previous study [33]. Briefly, 30 min after the last injection either of E4 alone or E4 combined with P4 and/or E2 or vehicle, animals passed through anesthesia with isoflurane (induction 3.0%; maintenance, 1.50%), and the left common carotid artery was double ligated and severed. The pups recovered for 1h with their dams. Then the pups passed through hypoxia in the special humidified hypoxic *in vivo* chamber (CoyLab, Grass Lake, MI, USA). For the first 20 min hypoxia was produced by decreasing oxygen concentration ( from 11% to 8 %) balanced with nitrogen, followed by hypoxia at a concentration of 8% o xygen balanced with 92% nitrogen for another 35 min at 37°C as previously described [33]. The sham group also passed through operation but the carotid artery was neither ligated nor severed and the pups were not exposed to hypoxia. Rat pups were euthanized at P14 (Figure 6A).

### Therapeutic (treatment) effect of E4 alone or E4 combined with E2 and/or P4 (Figure 6B)

To compare the therapeutic effect of E4 alone or E4 in combination with E2 and/or P4 after hypoxic-ischemic insult at P7, pups were designated to one of groups: sham group (*n* = 12), vehicle (*n* = 15), 5mg/kg/d E4 (*n* = 11), 10mg/kg/d E4 (*n* = 11), 5mg/kg/d E4+1.6mg/kg/d P4 (*n* = 11), 10mg/kg/d E4+1.6mg/kg/d P4 (*n* = 11), 5mg/kg/d E4+16mg/kg/d P4 (*n* = 11), 10mg/kg/d E4+16mg/kg/d P4 (*n* = 13), 5mg/kg/d E4+136ng/kg/d E2 (*n* = 11), 10mg/kg/d E4+136ng/kg/d E2 (*n* = 11), 5mg/kg/d E4+1.6mg/kg/d P4+136ng/kg/d E2 (*n* = 12), 10 mg/kg/d E4+1.6mg /kg/day P4+136ng/kg/d E2 (*n* = 11), 5mg/kg/d E4+16mg/kg/d P4+136ng/kg/d E2 (*n* = 11), 10mg/kg/d E4+16mg/kg/d P4+136ng/kg/d E2 (*n* = 12) groups. Estetrol concentrations 5mg/kg/d and 10mg/kg/d were the most successful doses used in our previous studies [33]. At P7, a model of hypoxia-ischemia in immature rats was used [42] with slight modifications as we already reported previously [33]. Briefly, animals passed through anesthesia with isoflurane (induction, 3.0%; maintenance, 1.5.0%) and the left common carotid artery was double ligated and severed in rat pups of the vehicle and treated by steroids groups. The pups recovered for 1h with their dams and then passed through hypoxia in the special humidified hypoxic *in vivo* chamber (CoyLab, Grass Lake, MI, USA). Hypoxia was produced by the decreasing concentrations of oxygen ( from 11% to 8 %) balanced with nitrogen for 20 min, and then by 8% oxygen balanced with 92% nitrogen for another 35min at 37°C as we previously reported [33]. Immediately after hypoxic ischemic insult rat pups were administered ip either vehicle or E4 alone or E4 combined with P4 and/or E2. The sham group was also anesthetized and operated but neither was exposed to HI operation nor injected. Rat pups were euthanized at P14 (Figure 6B).

### Measurement of rat pups rectal temperature

To determine the possible effect of E4 treatment alone or E4 combined with other steroids on rat pups body and brain temperatures the measurement of the temperature per rectum was done with a multiple thermometer (BAT-10R) and a specific RET-4 probe (Bio Medical Instruments, Zollnitz, Germany) after hypoxic insult at 0, 2, and 4h time points as described previously [33]. The variability of the rectal/body temperature was kept at low level by making the temperature measurements in a 25°C room [37]. It is well-known that the rectal temperature correlates positively with the brain temperature [100, 101]. The association between increased temperature and death or disability has at least 3 equally plausible explanations; brain injury raises body temperature, raised body temperature results in extension of the brain injury, or raised temperature is a marker for a process of which a part is encephalopathy [52].

### Brain and blood samples preparation

The pups were sacrificed at P14. Preparation of the brain and blood samples were performed according to our protocol already used previously [33]. Briefly, animals were deeply anesthetized. Blood serum samples were prepared and stored at −80°C. Transcardial perfusion of animals was performed with 0.9% saline solution followed by the perfusion of the 4% paraformaldehyde in PBS at 4°C. The brains, after being quickly removed, were weighed and fixed for 24h, followed by embedment in paraffin.

### The rat pups weight measurement

The rat pups postoperative well-being was monitored in pretreated (neuroprotective model) and treated (therapeutic model) groups. Body weights were measured from P7 to P14.

### Hematoxylin-eosin staining

Paraffin embedded brain samples were sliced into 5 μm-thick coronal sections at the hippocampus level [102]. Sections were deparaffinized and rehydrated and hematoxylin and eosin staining was performed.

### Intact cell number per visual field

Intact cell number was evaluated on 10 hematoxylin-eosin stained brain sections from each group. Counting was performed at magnification of 400x in 3 different fields of the respective brain areas in the cortex and different hippocampal regions: dentate gyrus (DG), subgranular zone (SGZ), cornu ammonis (CA1, CA2/CA3). The sections were analyzed as described in our previous study [33].

### MAP-2 staining

MAP-2 staining was performed on pups' coronal brain sections according to the protocol previously published [33]. Briefly, after antigen retrieval with citrate buffer, endogenous peroxidase activity was blocked, followed by second blocking and incubation with MAP-2 at a dilution of 1: 1000 (mouse monoclonal antibody ; Sigma, St. Louis, MI, USA). B iotinylated goat anti-mouse immunoglobulin G (Vector, Burlingame, CA, USA) addition was followed by the antibody detection by using the avidin-biotin complex method (Vector), with 3,3-diaminobenzidine (DAB).

The infarct area corresponds to a loss of MAP-2 staining. 10 samples from each study group of both study designs were analyzed as in our previous study [33]. The ratio of the MAP-2 positive area in sham operated animals was considered as 1.0 by default.

### Doublecortin and vascular endothelial growth factor double immunofluorostaining

To determine the effect of E4 alone or E4 combined with other steroids on neuro- and vasculogenesis in different regions of the hippocampus and the cortex, marker for neurogenesis doublecortin (DCX) and marker for angiogenesis-vascular endothelial growth factor (VEGF) were used as in previously published protocol [33]. Briefly, the sections proceeded through antigen retrieval step with citrate buffer and then endogenous peroxidase activity was blocked, followed by second blocking with normal goat serum and incubation with DCX 1:1000, and VEGF 1:100 (rabbit polyclonal antibody; Abcam, Cambridge, UK and mouse monoclonal antibody ; Abcam, Cambridge, UK respectively) overnight. Alexa fluor goat anti-rabbit 1:1000 and Alexa fluor goat anti-mouse 1:1000 (Invitrogen Inc., Life technologies; Gent, Belgium) were used as secondary antibodies. The sections were incubated and mounted with DAPI containing medium (Vector, Burlingame, CA, USA). 10 samples from each study group were analyzed as already described in our previous study [33]. The percentage of positively stained DCX and VEGF cells was calculated.

### ELISA to detect blood serum glial fibrillary acidic protein (GFAP) (brain damage marker)

ELISA for serum GFAP (USCNK Lifesciences Inc., China) was performed according to the manufacturers' recommendations.

### Statistical analysis

For statistical analysis was used the Statview statistics package (Abacus Concepts, Inc., Berkeley, CA, USA). For s tatistical comparisons was used ANOVA followed by Fisher's PLSD, Scheffe's and Bonferroni/Dunn post-hoc tests with P ≤ 0.05 considered as significant. All values are expressed as mean±SEM.

## SUPPLEMENTARY MATERIAL FIGURES


