# Supplementary material for: Use of estetrol with other steroids for attenuation of neonatal hypoxic-Ischemic brain injury: to combine or not to combine?
Source: Oncotarget. 2016 May 26;7(23):33722–43. doi: 10.18632/oncotarget.9591 (PMC5085115; doi:10.18632/oncotarget.9591)
Supplement: Supplementary file 1 [file oncotarget-07-33722-s001.pdf]

Use of Estetrol with other Steroids for Attenuation of Neonatal Hypoxic-Ischemic brain injury: to combine or not to combine?

Supplementary Material

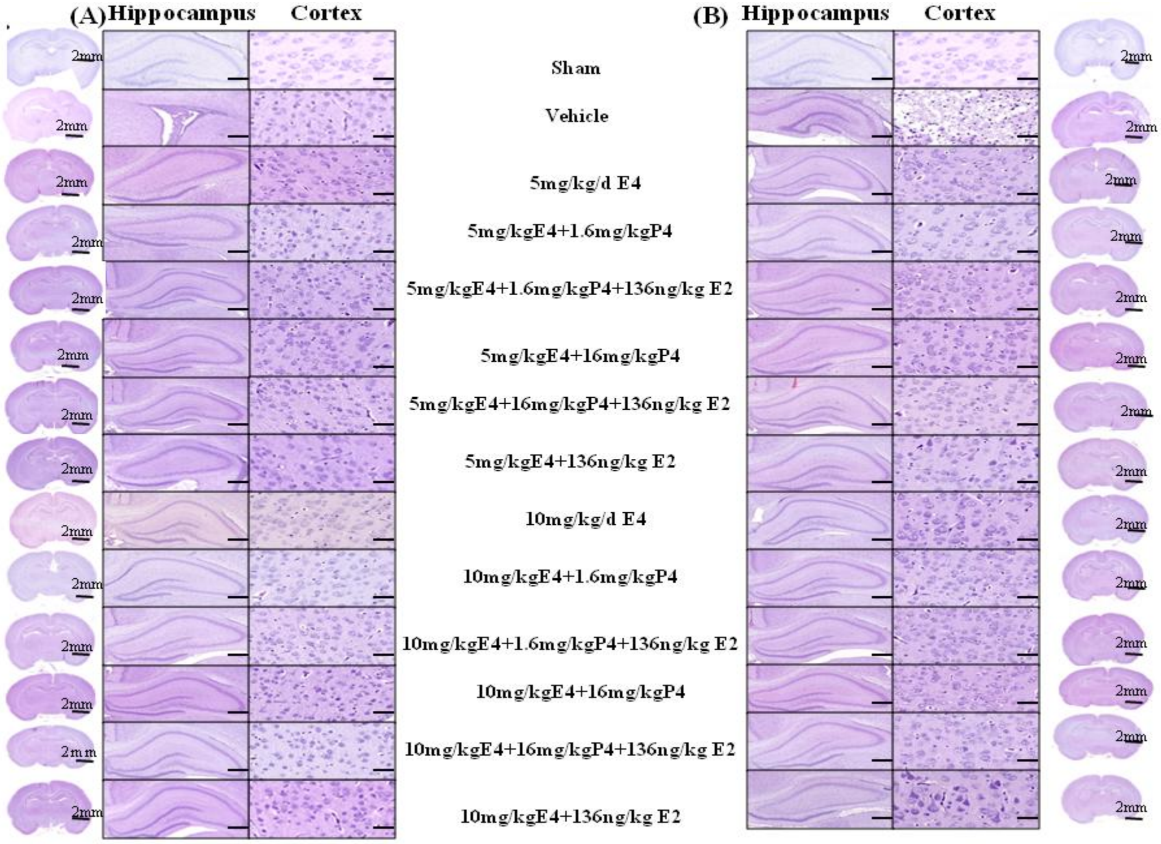

Supplementary Figure 1. Representative views of Hematoxylin-eosin-stained brain coronal sections from rat pups pretreated/treated by E4 alone or in combination with P4 and/or E2.

Paraffin embedded brain samples were sliced into 5 µm-thick coronal sections at the hippocampus level. Sections were deparaffinized and rehydrated and hematoxylin and eosin staining was performed. Brain coronal sections (Scale bar: 2mm) with hippocampus region

(Scale bar: 500µm), and the cortex (Scale bar: 100µm) from pretreated **(A)** and treated **(B)** study groups are presented.

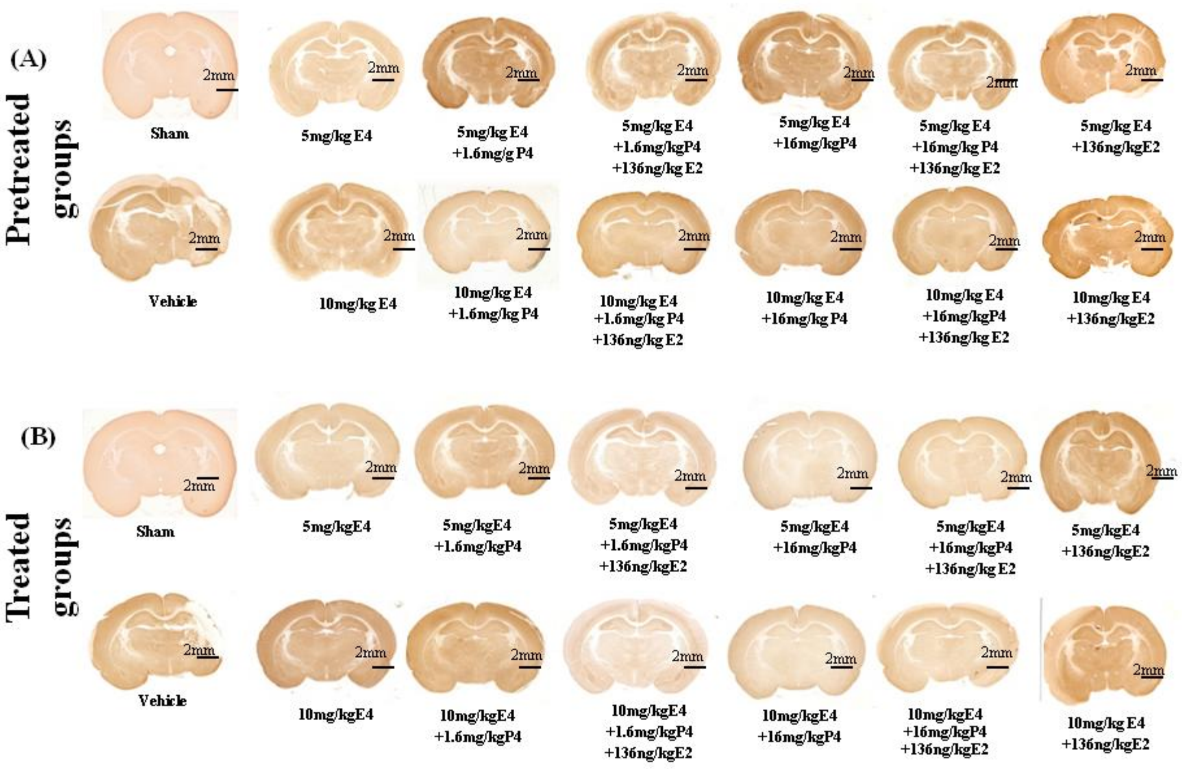

Supplementary Figure 2. Representative views of MAP2-stained coronal brain sections from groups pretreated or treated with E4 alone or in combination with P4 and/orE2.

From left to right are presented MAP2 stained sections from pretreated **(A)** and treated **(B)** groups. In sections from the vehicle pretreated/treated animals was observed an existence of MAP2 negatively stained areas in the hippocampus and the cortex at the left, damaged side. Scale bar: 2 mm.
